# The role of intellectual humility leadership on thriving at work and performance of new generation employees

**DOI:** 10.3389/fpsyg.2025.1673728

**Published:** 2025-09-19

**Authors:** Changchun Gao, Aiwen Niu, Chenhui Yu

**Affiliations:** Glorious Sun School of Business and Management, Donghua University, Shanghai, China

**Keywords:** intellectual humility, positive work attitude, core self-evaluation, thriving at work, work performance, JD-R theory, social exchange theory, SEM

## Abstract

**Background:**

As career maturity and self-awareness increase among new-generation employees, they seek not only material rewards but also well-being and meaningful work.

**Methods:**

Drawing on Job Demands–Resources (JD-R) theory and Social Exchange Theory (SET), this study establishes a moderated mediation model to examine how intellectual humility leadership influences thriving at work and job performance, with positive job attitudes as a mediator and core self-evaluation (CSE) as a moderator. Data from 518 manager–subordinate dyads in Chinese SMEs were analyzed using structural equation modeling (SEM).

**Results:**

The findings show that intellectual humility leadership enhances thriving and performance by fostering positive job attitudes, which mediate these effects. Moreover, CSE moderates this relationship: the positive effect of intellectual humility leadership on attitudes is stronger when CSE is high and weaker when it is low.

**Conclusion:**

The study contributes theoretically by identifying intellectual humility leadership as a critical job resource and relational signal, refining JD-R and SET, and highlighting generational variations in leadership effectiveness. Practically, it suggests cultivating intellectual humility in leadership development to strengthen engagement, performance, and well-being among Millennials and Gen Z. Limitations include the SME focus and survey design; future research should test additional mediators and adopt longitudinal or mixed-method approaches.

## 1 Introduction

In modern society, globalization, marketization, and digitalization continuously challenge traditional values, with instrumental rationality increasingly prevailing over value rationality. As a result, although the new generation of employees contributes substantially to organizational economic value, they often struggle to attain a corresponding sense of well-being. Philosophical inquiries have examined this phenomenon from the perspectives of the “society of fatigue,” “meritocracy,” and “nihilism” (Han, 2021; Sandel, 2020; Geertz, 1984). However, within the field of organizational behavior, theoretical explanations and empirical investigations on this issue remain limited. In this context, intellectual humility provides a valuable perspective to fill this gap. Traditionally regarded as a moral virtue, intellectual humility has increasingly been recognized as a cognitive virtue in contemporary scholarship. Baehr argues that virtues can have intellectual dimensions, and intellectual humility encourages individuals to be effective epistemic agents, akin to the cognitive counterpart of moral humility (Baehr, 2011). Samuelson et al. (2015) defines it as a balanced position between the extremes of intellectual arrogance (claiming more knowledge than warranted) and intellectual diffidence (claiming less knowledge than warranted), while Porter et al. (2022) describe it as a metacognitive ability to recognize the limitations of one’s knowledge and beliefs. Intellectual humility is further explored by scholars such as Danovitch et al. (2019), who examine its cognitive dimensions and its relationship with knowledge acquisition. Despite the growing practical significance of intellectual humility, no consensus definition has yet been established. This is largely because philosophers and psychologists pursue different theoretical objectives in their research: philosophers emphasize its normative implications, whereas psychologists focus more on its operationalization and measurement.

Based on the above theoretical basis, scholars have further proposed the concept of intellectual humility leadership. Leary et al. (2017) defined it as one’s beliefs may be flawed, while appropriately recognizing the limitations of the evidence base for those beliefs and the limitations in acquiring and evaluating relevant information. This humility attitude helps establish a trusting and cooperative team atmosphere, where leaders prefer to collaborate with team members and leverage collective wisdom for success. Compared with related constructs such as general humility leadership, intellectual humility leadership possesses a distinctive cognitive core. General humility leadership (also called humble leadership) primarily emphasizes humility at the moral and interpersonal levels, such as admitting mistakes, recognizing others’ contributions, and reducing self-centeredness (Chan et al., 2024). In contrast, intellectual humility leadership highlights epistemic qualities, namely acknowledging cognitive limitations, maintaining openness, and revising one’s views in light of new evidence. This distinction underscores the theoretical value of intellectual humility leadership as an emerging construct in leadership research. Although research on intellectual humility leadership is still in its early stages, accumulating evidence has demonstrated its associations with a range of employee- and organization-level outcomes, including job satisfaction, organizational commitment, organizational citizenship behavior, and work performance (Porter et al., 2022). However, existing studies have largely focused on the general employee population, with limited attention given to the new generation workforce. As the main force in today’s workplace, Millennials (born between 1981 and 1996) and Generation Z (born in 1997 and thereafter) differ markedly from older generation in their values and work expectations, placing greater emphasis on autonomy, meaningfulness, and psychological alignment (Gasiorowski, 2023). Without systematic research targeting this group, it is difficult to fully understand the unique mechanisms through which intellectual humility leadership enhances their job satisfaction and performance.

In organizational research, work well-being in the traditional sense is generally understood as the satisfaction and pleasure employees derive from their work (Chingan Thottathil and Nandakumar, 2025). This encompasses comfort with the work environment, perceived fairness of income, and positive emotional experiences. It primarily emphasizes employees’ affective fulfillment and emotional stability. However, for Millennials and Generation Z, this static notion of well-being is no longer sufficient to capture their needs. They place greater emphasis on maintaining vitality and growth at work, often referred to as thriving at work. Thriving at work represents a positive psychological state in which employees simultaneously experience a sense of energy and continuous learning (Spreitzer et al., 2005). Compared with traditional well-being, it more fully reflects new-generation employees’ pursuit of meaningfulness, self-development, and accomplishment (Tian and Li, 2024). Prior studies have shown that thriving at work not only enhances employees’ work engagement and creativity but is also closely linked to long-term performance and organizational sustainability. Based on the Job Demands–Resources (JD-R) theory, which analyzes how work environments impact well-being and performance (Bakker and Demerouti, 2017), jobs are typically categorized into job demands and job resources. Intellectual humility leadership can be conceptualized as a critical job resource that alleviates stress caused by job demands by providing psychological safety, emotional support, and learning opportunities, thereby stimulating employees’ positive motivation (Niu et al., 2025). Accordingly, intellectual humility leadership is expected to serve as an important leadership style for fostering thriving at work among new-generation employees.

Thriving at work primarily reflects employees’ psychological and developmental states, whereas performance concerns the achievement of organizational goals. Although those two concepts are related, they are not equivalent. Therefore, the role of intellectual humility leadership in enhancing performance also warrants in-depth investigation. Social Exchange Theory (SET) emphasizes that favorable treatment from leaders engenders employees’ reciprocal attitudes and behaviors (Raziq et al., 2025). Within this framework, leaders’ respect, openness, and support are perceived by employees as signals of positive investment, which in turn elicit higher levels of responsibility and performance (Casimir et al., 2014). For new-generation employees, such interactions not only fulfill their needs for meaning and growth at work, thereby fostering stronger thriving at work, but also, from an organizational perspective, translate into greater efficiency and performance outcomes. In other words, intellectual humility leadership enhances thriving at work by addressing employees’ psychological needs, while simultaneously improving organizational performance by promoting cooperation and responsibility, thus achieving a win–win situation for both employees and organizations.

Intellectual humility leadership can satisfy employees’ psychological growth needs while also enhancing organizational performance, but this dual effect does not occur directly; rather, it relies on specific psychological mechanisms. This study introduces positive job attitudes as a mediating variable, as they not only reflect employees’ emotional and cognitive orientations (e.g., work engagement, organizational commitment, and positive affect) but also simultaneously link thriving at the individual level with performance at the organizational level. Positive work attitudes refer to employees’ affective and cognitive orientations formed in the workplace, typically manifested in high levels of work engagement, organizational commitment, and positive emotional experiences (Susanty and Miradipta, 2013). In contrast, variables such as psychological safety, trust, or leader–member exchange are more oriented toward relational or climate aspects and do not symmetrically cover both outcomes. From an integrated perspective of the JD-R and SET frameworks, this study argues that intellectual humility leadership fosters supportive environment that provide psychological and resource security, while also building reciprocal relationships that strengthen employees’ sense of belonging and responsibility, thereby creating a win–win situation for employees and organizations through positive job attitudes.

According to the JD-R theory, individual differences shape how employees perceive and use leadership as a job resource (Tummers and Bakker, 2021). Prior research has shown that traits such as emotional states, work engagement, or psychological safety can moderate leadership effects, but these state-like characteristics are often unstable and context-dependent (Taşkan et al., 2024; Changar and Sesen, 2025; Erkutlu and Chafra, 2016). In contrast, CSE is a stable personality trait reflecting individuals’ beliefs about their competence, worth, and control. First proposed by Judge et al. (1997), CSE refers to individuals’ fundamental evaluations of their own abilities and worth, consisting of four dimensions: self-esteem, generalized self-efficacy, locus of control, and neuroticism. As a higher-order personal resource, CSE largely determines how employees view and respond to their external environment (Kim et al., 2015). This stability makes CSE a solid boundary condition for leadership effectiveness. Within the JD-R framework, CSE is considered a key personal resource that shapes employees’ psychological reactions and resource mobilization when facing leadership behaviors (Kim and Beehr, 2020). Specifically, employees with low levels of CSE rely more on the cognitive support and psychological safety provided by leaders and thus benefits more from intellectual humility leadership; whereas employees with high level of CSE draw on their intrinsic confidence and sense of control to maintain positive attitudes, making them relatively less dependent on leadership, thereby attenuating leadership effects. Therefore, CSE plays a critical moderating role in the relationship between intellectual humility leadership and positive work attitudes.

## 2 Theoretical framework and hypothesis development

### 2.1 Intellectual humility leadership and positive work attitude

Organizations can enhance their performance by influencing employees’ job attitudes and by instituting management practices that support the development of intrinsic motivation (Sandel, 2020). Judge and Kammeyer-Mueller (2012) define job attitudes as “evaluations of one’s job that express one’s feelings, beliefs, and attachment to it.” According to Bagozzi (1992), the term “attitude” encompasses preferences, emotions, beliefs, expectations, judgments, evaluations, values, opinions, and intentions. Job attitudes can be either positive or negative, and since attitudes typically predict behavior (Ajzen and Fishbein, 1980), they serve as an important indicator of behavioral antecedents. Employees’ job attitudes are shaped by both internal and external factors: internal factors include work-related values, self-efficacy, trust, and career development (Aâ, 2013; McNatt and Judge, 2008; Mathew and Zacharias, 2016; Rebeka and Indradevi, 2015); external factors include relationships with colleagues, leadership styles, and organizational policies (Ahmad et al., 2020; Khuwaja et al., 2020; Noah and Steve, 2012).

Intellectual humility (abbreviated as IH) is a leadership quality that encompasses both humility and intellectual acumen (Krumrei-Mancuso and Begin, 2022). In psychology, it reflects leaders’ awareness of their own limitations. In interpersonal interactions, IH has been associated with a range of positive and prosocial qualities, such as agreeableness, openness, perspective-taking, helpfulness, generosity, and high-quality social relationships (Porter et al., 2021). Integrating Job Demands–Resources (JD-R) theory and Social Exchange Theory (SET), this study argues that intellectual humility leadership promotes positive job attitudes through two mutually reinforcing mechanisms. From the JD-R perspective, intellectual humility leadership functions as a key job resource by providing employees with psychological safety, emotional support, and cognitive openness, thereby buffering the stress caused by job demands and stimulating positive motivation (Niu et al., 2025; Krumrei-Mancuso and Begin, 2022). Intellectual humility can be viewed as a signal of fairness and support (Karabegovic and Mercier, 2024). From the perspective of SET, such signals are likely to initiate reciprocal processes, encouraging employees to respond with positive emotions, stronger commitment, and constructive attitudes. Accordingly, intellectual humility leadership operates through dual pathways—as a “job resource” and as a “social signal”—to promote positive job attitudes via the combined effects of resources and reciprocity.

Based on the above discussion, this study proposes Hypothesis 1 (H1):

*Hypothesis 1*: There is a positive relationship between intellectual humility leadership and employees’ positive work attitudes.

### 2.2 Intellectual humility leadership, thriving at work and work performance of new generation employees

Research has shown that thriving at work can be viewed as a form of “happy productivity,” bringing profound positive changes to both organizations and individuals. For example, a piece-rate experiment demonstrated that happiness increased participants’ productivity by 12%, while another study found that employees who reported being happy achieved an average increase of 37% in sales performance (Oswald et al., 2009; Sgroi, 2015). From the perspectives of employee retention and organizational competitiveness, ensuring employees’ thriving at work is an important managerial task. Despite the significance of enhancing thriving among new-generation employees, many firms still treat it as a marginal issue. Spreitzer et al. (2005) first introduced the concept of thriving at work, defining it as a psychological state in which individuals simultaneously experience vitality and learning. Every employee has the potential to thrive at work, and this potential can be activated and mobilized through leadership and other contextual factors. Carmeli and Spreitzer (2009) further argued that thriving at work is essentially a positive subjective experience that enables individuals to perceive growth and progress in their work. For new-generation employees, who emphasize self-development, learning, and self-actualization, thriving at work is a critical indicator of both perceived work value and well-being.

Every employee has the potential to thrive at work, and this potential can be activated and mobilized through leadership and other contextual factors. Previous studies have extensively examined the impact of different leadership styles on employees’ thriving at work, but relatively few have approached it from the perspective of intellectual humility leadership. By fostering an atmosphere of trust and support (Johnson, 2022), it reduces the stress caused by job demands and provides employees with psychological safety and opportunities for growth. Such resources not only buffer the negative emotions experienced by new-generation employees in high-pressure contexts but also stimulate vitality and learning motivation, thereby significantly enhancing thriving at work. For Millennials and Generation Z in particular, who attach greater importance than older generations to autonomy and continuous learning opportunities (Mantha and Krishna, 2024), the openness, support, and egalitarian nature of intellectual humility leadership closely match their psychological needs. Therefore, this study proposes the following Hypothesis 2 (H2):

*Hypothesis 2*: There is a positive relationship between intellectual humility leadership and the thriving at work of the new generation employees.

Leaders who exhibit intellectual humility—by acknowledging their limitations, inviting input, and interacting fairly—demonstrate behaviors that can be interpreted as signals of fairness and support, as established earlier (Karabegovic and Mercier, 2024). From the perspective of SET, employees interpret such signals as evidence of a high-quality exchange relationship. In return, they are motivated to reciprocate by demonstrating stronger engagement, heightened responsibility, and enhanced performance (Ivziku et al., 2025). New-generation employees are often uncomfortable with rigid hierarchies and instead seek to realize their self-worth in open and egalitarian environments. In particular, they value having a “voice” in organizations and expect their opinions to be heard and respected (Thomas et al., 2025). Intellectual humility leadership reduces power distance, encourages voice, and recognizes contributions, thereby increasing their sense of responsibility and organizational commitment (Porter and Schumann, 2018). Consequently, they are more willing to reciprocate with high performance, achieving a win–win outcome for both individuals and organizations. Therefore, this study proposes the following Hypothesis 3 (H3):

*Hypothesis 3*: There is a positive relationship between intellectual humility leadership and employees’ work performance.

### 2.3 The mediating role of positive work attitudes

Spreitzer et al. (2005) also constructed a theoretical model of thriving at work, explaining how individual characteristics (e.g., knowledge level, positive emotions), relational characteristics (e.g., support and trust), contextual characteristics (e.g., job autonomy, climate of trust), and agentic work behaviors (e.g., task focus and exploration) jointly contribute to thriving. Kleine et al. (2019) provided empirical evidence that thriving at work positively influences job attitudes. However, from both theoretical and empirical perspectives, positive job attitudes can also significantly shape thriving at work. For new-generation employees in particular, who seek psychological alignment, freedom of expression, and value recognition, positive job attitudes—such as job satisfaction, organizational commitment, and optimism about career prospects—reinforce their sense of meaning and autonomy at work, thereby further enhancing thriving.

In summary, positive work attitudes enable employees to gain a stronger sense of satisfaction, achievement, and positive emotions, thereby enhancing their thriving at work. Therefore, this study proposes the following Hypothesis 3 (H4):

*Hypothesis 4 (H4)*: Positive work attitudes of new-generation employees are positively related to their thriving at work.

As previously discussed, intellectual humility leadership can be regarded as a critical job resource. Based on the JD-R framework, when leaders demonstrate openness, respect, and psychological safety, employees are more likely to develop positive job attitudes (Dhaneesh et al., 2025). Such attitudes buffer the negative effects of job demands and stimulate new-generation employees’ vitality, motivation to learn, and proactivity, thereby enhancing their thriving at work. Based on Hypotheses 1 and 4, it can be inferred that intellectual humility leadership indirectly influences employees’ thriving at work through positive work attitudes. In other words, intellectual humility leadership directly enhances employees’ thriving at work while also indirectly improving it by fostering positive work attitudes. Based on this reasoning, this study proposes Hypothesis 5 (H5):

*Hypothesis 5 (H5)*: Positive work attitudes of new-generation employees mediate the relationship between intellectual humility leadership and thriving at work.

Research has also revealed a close relationship between job performance and the formation of positive job attitudes (Gagné and Deci, 2005; Ogbonnaya et al., 2017). Yet performance-contingent pay may exacerbate work pressure, potentially undermining the positive effects of such attitudes. Unfortunately, these studies have not treated positive attitudes themselves as stimuli in explaining their mechanisms of influence. According to SET, employees’ positive attitudes and behaviors are shaped by organizational rewards and support (Tan et al., 2025). Such favorable exchange relationships stimulate employees’ engagement and enthusiasm, thereby fostering thriving at work. Positive job attitudes (e.g., high job satisfaction, strong organizational commitment, and career confidence) improve employees’ psychological states and work motivation, prompting them to demonstrate greater concentration and involvement at work (Herman, 2013; Halepota, 2011). Furthermore, employees with positive job attitudes are more likely to proactively seek opportunities for learning and growth, thereby enhancing their career development potential and work experience (Weer and Greenhaus, 2020). For new-generation employees, who prioritize meaningful work and growth-driven careers, positive job attitudes not only represent a response to organizational support but also constitute a central pathway for realizing self-worth and professional advancement.

In summary, positive work attitudes drive employees to concentrate more fully on their tasks, improve performance, and create greater organizational value. Therefore, this study proposes Hypothesis 6 (H6):

*Hypothesis 6 (H6)*: Positive work attitudes of new-generation employees are positively related to their job performance.

According to Social Exchange Theory (SET), employees interpret leaders’ openness and respect as signals of support and reciprocate through the development of positive work attitudes (Ko and Hur, 2014). These attitudes, in turn, enhance employees’ sense of responsibility and engagement, thereby improving job performance. Based on Hypotheses 1 and 6, this study proposes Hypothesis 7 (H7):

*Hypothesis 7 (H7)*: Positive work attitudes of new-generation employees mediate the relationship between intellectual humility leadership and job performance.

### 2.4 The moderated mediation role of employees’ core self-evaluation

Judge et al. (1997) first proposed the concept of core self-evaluation (CSE), defining it as an individual’s fundamental appraisal of their own abilities and worth. CSE is regarded as a higher-order personality trait composed of four specific dimensions: self-esteem, emotional stability (the inverse of neuroticism), locus of control, and generalized self-efficacy (Judge et al., 2003). Self-esteem reflects the overall affirmation of one’s value; emotional stability represents the capacity for emotional self-regulation; locus of control refers to the degree to which individuals believe they can control life events; and self-efficacy denotes confidence in one’s ability to accomplish tasks (Hatter, 1990; Bandura, 1982; Goldberg, 1990; Rotter, 1966). Previous studies often examined these four dimensions separately, revealing their associations with work outcomes. However, focusing on single dimensions can only capture partial aspects of psychological resources and fails to provide a comprehensive understanding of individuals’ evaluations of themselves and their environment. In contrast, CSE, as an integrative construct, surpasses the explanatory power of single traits and provides a more systematic prediction of individuals’ attitudes and behaviors at work.

Both domestic and international studies have confirmed the relationship between core self-evaluations (CSE) and employees’ attitudes and behavioral outcomes. For example, a meta-analysis by Judge and Bono (2001) indicated that CSE is a strong predictor of job satisfaction across different organizational contexts. Other scholars, using cross-validation, have demonstrated its significant effects on job satisfaction, task performance, and job burnout. Moreover, research focusing on new-generation employees has shown that higher levels of CSE are associated with greater enthusiasm and engagement at work (Li and Ding, 2022; Zhang and Du, 2011). However, most of the existing studies have primarily concentrated on the direct effects of CSE on variables such as job satisfaction, burnout, and engagement, or explored its mediating role in different groups, while relatively little attention has been paid to its boundary role in the mechanisms of leadership styles.

On this basis, leadership research has further revealed the important role of core self-evaluations in leader–employee interactions. Most studies on leadership and performance regard subordinate performance as the result of the interaction between leaders and employees, focusing on the causal role in this process. Given that effective leaders can enable followers to realize their optimal capabilities (Bass and Bass, 2008), leaders can amplify the positive relationship between followers’ CSE and their outcomes. High-CSE individuals also expect to establish high-quality relationships with their superiors within the organization. Some scholars have examined the relationship between transformational leadership, servant leadership, authentic leadership, and CSE (Chai et al., 2017). Although these studies investigated different dependent, mediating, and moderating variables, they all confirmed the positive correlation between these leadership styles and CSE, with servant leadership and CSE being mutually influential (Resick et al., 2009; Schmidt, 2008; Rodríguez-Carvajal et al., 2010). However, these studies have mostly focused on traditional leadership styles, and relatively little attention has been paid to how the emerging concept of intellectual humility leadership influences core self-evaluations and their subsequent outcomes. Intellectual humility leadership helps build positive leader–employee relationships and provides a supportive work environment, thereby shaping employees’ CSE and work attitudes (Krumrei-Mancuso and Begin, 2022). As a job resource, intellectual humility leadership improves the work context by offering support, autonomy, and psychological safety.

This mechanism can be further explained within the framework of the Job Demands–Resources (JD-R) theory. According to JD-R theory, individuals in the workplace rely not only on external job resources but are also constrained by internal personal resources (Bakker and Demerouti, 2007). Core self-evaluation, as a stable personality trait, essentially represents a critical personal resource that determines how employees perceive and utilize the external resources provided by leaders (Tummers and Bakker, 2021). High levels of CSE enhance employees’ ability to recognize and absorb the support, autonomy, and psychological safety conveyed through intellectual humility leadership, thereby strengthening the translation of such resources into positive work attitudes. In contrast, low levels of CSE restrict this resource utilization, thereby weakening the effectiveness of leadership. Thus, CSE functions as a boundary condition in the mechanism through which intellectual humility leadership influences employee outcomes, a logic consistent with the JD-R theory’s explanation of the interaction between personal and job resources.

In the context of intellectual humility leadership, the differences in employees’ levels of core self-evaluation (CSE) become particularly salient. Employees with high CSE possess stronger self-esteem, self-efficacy, and emotional regulation, making them more likely to perceive the respect and understanding conveyed by their leaders and to actively absorb the support, autonomy, and psychological safety provided. The interaction of these external resources with their internal resources produces an “amplification effect,” which further strengthens the positive influence of leadership on work attitudes and performance (Leary, 2022; Piwowar-Sulej and Iqbal, 2025; Neves and Champion, 2015). In contrast, employees with low CSE often lack the confidence or capability to rely on their own resources to translate positive attitudes into sustained performance. However, the support and care offered by intellectual humility leadership can generate a “compensatory effect,” partially offsetting their lack of internal resources, helping them develop positive work attitudes, and mitigating the negative impact of low CSE on work outcomes.

From the perspective of employees, a positive work attitude can stimulate vitality and engagement, but whether it can be sustained and further develop into thriving at work depends on individuals’ internal psychological resources (Xanthopoulou et al., 2007). Employees with high CSE are able to amplify the positive effects of leadership (Newman et al., 2018), as they typically possess stronger emotional regulation and self-motivation, which enable them to extend positive attitudes into sustained energy, learning willingness, and resilience, thereby demonstrating higher levels of thriving at work. In contrast, employees with low CSE may experience short-term improvements supported by positive attitudes, but due to a lack of stable psychological resources, they find it difficult to maintain high levels of engagement. Therefore, core self-evaluation moderates the indirect effect of intellectual humility leadership on thriving at work through positive work attitudes.

Based on the above arguments, and in line with Hypotheses 5 and 7, this study proposes Hypotheses 8a and 8b:

*Hypothesis 8a (H8a)*: Core self-evaluation moderates the indirect effect of intellectual humility leadership on job performance through positive work attitudes. Specifically, when core self-evaluation is higher, it strengthens the relationship between intellectual humility leadership and positive work attitudes.

*Hypothesis 8b (H8b)*: Core self-evaluation moderates the indirect effect of intellectual humility leadership on thriving at work through positive work attitudes. Specifically, when core self-evaluation is higher, it strengthens the relationship between intellectual humility leadership and positive work attitudes.

**Figure 1:**
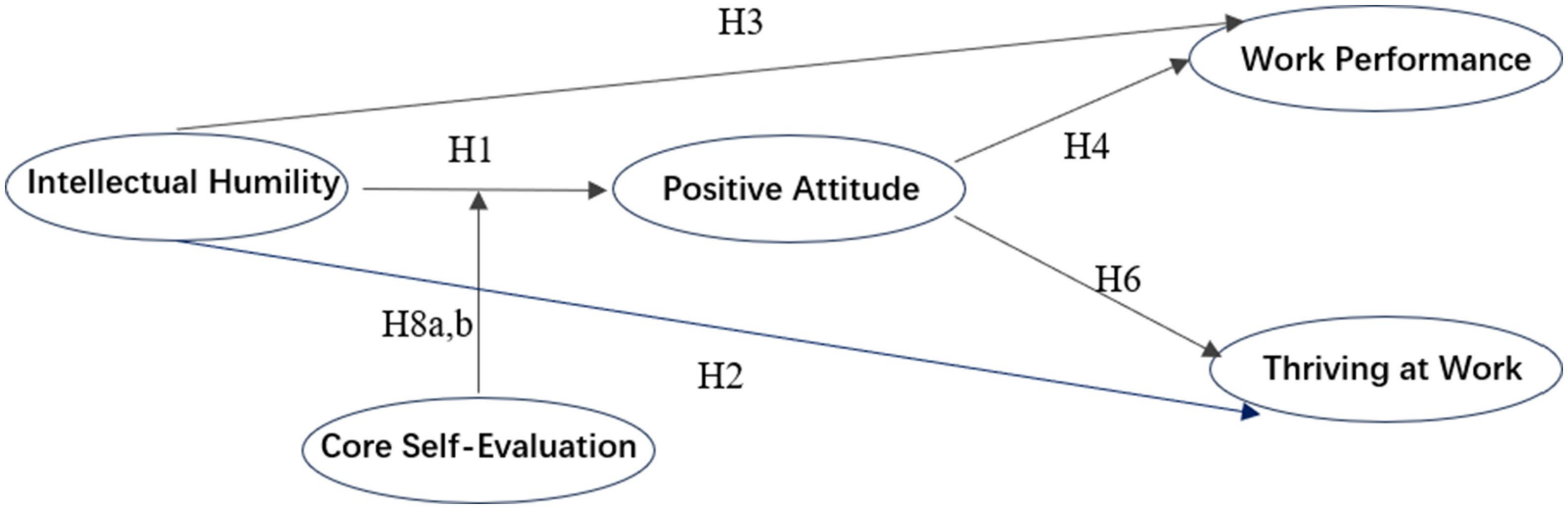
Hypothesized research model.

Based on the above hypotheses, the hypothesized research model is presented in Figure 1.

## 3 Research design

### 3.1 Survey process and participants

The data collection for this study took place from May 2022 to May 2023. The sample selection criteria focused on small and medium-sized enterprises (SMEs) that had been established for more than 2 years. The survey was conducted using electronic questionnaires, targeting middle and senior managers of SMEs and their subordinates. For convenience, some regions utilized pre-sampling frameworks through university MBA (EMBA) centers and EDP centers to obtain email addresses of corporate executives. These executives were first asked to rate items related to the independent variable—intellectual humility in leadership. Subsequently, their subordinates were invited to answer questions related to the dependent variable, mediating variable, and moderating variable.

The questionnaire design involved translation and back-translation between Chinese and English, ensuring no semantic discrepancies. A pre-survey was conducted using 50 randomly selected questionnaires, and adjustments were made based on the results. To avoid common method bias, data were collected in two periods. The first period, from May to July 2022, involved 10 participants who answered questions on the moderating variable, dependent variable, and control variables. The second period, from August to October 2022, involved 20 participants: 10 answered questions on the independent variable, and 10 on the mediating variable. Participants in both waves were required to answer control variable questions. This staggered approach reduced endogeneity concerns related to reverse causality and omitted variables.

In total, 869 managers agreed to participate in the survey. While the managers completed the section on intellectual humility, they distributed the remaining sections to at least one of their subordinates, who filled out the rest of the questionnaire. After excluding invalid questionnaires with obvious errors and missing data, as well as those from employees over 43 years old (since the study focused on new-generation employees), 518 valid questionnaires remained. The effective response rate was 59.61%.

The gender distribution of the participants (Gender) was 47.49% female and 52.51% male. In terms of education background (EB), 14.86% had a high school education or below, 30.89% had an associate degree, 41.7% had a bachelor’s degree, and 12.55% had a master’s degree or higher. The age characteristics (AGE) showed that the average age of leaders was 47.7 years, while the average age of new-generation employees was 33.1 years. Regarding tenure (years of work experience, YWE), the majority of participants had been in their positions for less than 1 year (61.59%), followed by 1–2 years (29.73%), and more than 2 years (8.69%). The industry affiliation (IA) of the participants included 25.32% in manufacturing, 20.18% in information technology, 15.47% in finance, 13.84% in services, 10.26% in education and research, 8.94% in healthcare, and 5.99% in retail.

### 3.2 Measures

For the adopted foreign research scales, this study employed a translation-back-translation procedure to avoid the influence of semantic differences. The questionnaires all used a 5-point Likert scale, ranging from 1 (strongly disagree) to 5 (strongly agree) to represent different levels.

(1) Positive attitude in workplace (PA): We measured Positive attitude (PA) in work place using the Arifin (2020)’s scale. Only items with factor loadings above 0.68 from the original scale were selected, such as “Your satisfaction with opportunities to develop your skills.”

(2) Core self-evaluation (CSE): We evaluated core self-evaluation in the workplace using a 12-item scale developed by Judge et al. (2003). An example item is ‘I am confident in achieving the goals I set for myself.’ The Chinese translation of this scale has been validated in articles on work values, such as Hou et al. (2014), making it suitable for measuring core self-evaluation among employees in Chinese contexts.

(3) Thriving at work (TaW): We measured thriving at work with a scale from Porath et al.’s (2012) literature, focusing on items that retained high factor loadings. The scale includes items such as “Today, I feel I am very productive in my learning.”

(4) Intellectual humility leadership (IH): We employed a scale developed by Leary to gauge intellectual humility leadership (2017), which includes 6 items like “I question my own views, positions, and opinions because they may be wrong; I reconsider my views when presented with new evidence.”

(5) Work Performance (WP): We used a work performance scale developed by Han (2006) to measure various performance outcomes, including task, relational, learning, and innovative performance. High factor loading items include “Even when supervisors are not present, I follow instructions.”

(6) Control variables: We included gender, age, education background, industry affiliation, and years of work experience as control variables. These variables are chosen based on their association with organizational behavior and employee psychological responses, as previous research has shown their impact on employees’ sense of thriving at work and work performance (Meng et al., 2011).

Data processing for this study was conducted using SPSS 26.0 and AMOS 28.0 software. Results from the Harman’s single-factor test showed that the first principal component explained 39.59% of the variance, indicating no serious common method bias. The variance inflation factors (VIF) were 1.371, 2.273, and 2.001, all of which are less than 5, suggesting that there is no multicollinearity problem in this study’s questionnaire. The Cronbach’s alpha coefficients for all variables exceeded 0.83, indicating good reliability. The study utilized scales from mature instruments sourced from domestic and international literature, ensuring content validity of the questionnaire. As Table 1 shows, standardized factor loadings for all items were above 0.76, demonstrating good convergent validity. Composite reliabilities (CR) for each variable exceeded 0.83, and average variance extracted (AVE) values were above 0.58, indicating high levels of convergent validity for the questionnaire.

**Table 1:** Question items and reliability tests for variables.

Variant	Measurement items	Playlods
IH (*α* = 0.895, AVE = 0.589 CR = 0.896)	I question my own opinions, positions, and viewpoints because they could be wrong.	0.784
	I reconsider my opinions when presented with new evidence.	0.744
	I recognize the value in opinions that are different from my own.	0.785
	I accept that my beliefs and attitudes may be wrong.	0.803
	In the face of conflicting evidence, I am open to changing my opinions.	0.752
	I like finding out new information that differs from what I already think is true.	0.735
CSE (*α* = 0.923, AVE = 0.602 CR = 0.924)	I am confident I get the success I deserve in life.	0.761
	Sometimes I feel depressed(r).	0.759
	When I try, I generally succeed.	0.762
	I complete tasks successfully.	0.803
	Overall, I am satisfied with myself.	0.772
	I determine what will happen in my life.	0.767
	I do not feel in control of my success in my career(r).	0.800
	I am capable of coping with most of my problems.	0.779
PA (*α* = 0.928, AVE = 0.683 CR = 0.928)	How satisfied are you with the opportunity to develop your skills.	0.829
	How satisfied are you with the work itself.	0.831
	I share many of the values of my organization.	0.836
	I am proud to tell people who I work for.	0.831
	Managers here can be relied upon to keep to their promises.	0.812
	Managers here deal with employees honestly.	0.818
WP (*α* = 0.869, AVE = 0.625 CR = 0.869)	Follows orders even when higher management is not present.	0.792
	Completes work assignments as required by formal performance appraisals.	0.802
	Value learning to gain experience and improve efficiency.	0.788
	Apply knowledge gained to solve problems encountered in the workplace.	0.779
TaW (*α* = 0.833, AVE = 0.636 CR = 0.835)	Today, I feel like I’m being productive.	0.768
	I am experiencing rapid growth.	0.788
	I see myself improving.	0.820

The abbreviations mentioned above refer to Intellectual Humility Leadership (IH), Core Self-Evaluation (CSE), Positive Attitude in Workplace (PA), Thriving at Work (TaW), and Work Performance (WP).

### 3.3 Results

In this study, confirmatory factor analysis (CFA) was employed to examine the discriminant validity among variables, and the results are shown in Table 2. According to Table 2, the five-factor model demonstrated the best fit: ( $\chi^{2}/\mathrm{df}$ = 1.286; RMSEA = 0.024, SRMR = 0.024, CFI = 0.990, TLI = 0.989, AGFI = 0.934). Moreover, these fit indices significantly outperformed those of other models, indicating good discriminant validity among the variables.

**Table 2:** Confirmative factor analysis.

Variables	χ^2^/df	RMSEA	SRMR	AGFI	TLI	CFI
Five-factor model	1.286	0.024	0.024	0.934	0.989	0.990
Four-factor model^a^	11.736	0.144	0.300	0.379	0.590	0.629
Three-factor model^b^	6.562	0.104	0.100	0.586	0.788	0.806
Two-factor model^c^	11.415	0.142	0.152	0.375	0.602	0.634
Single-factor model	3.043	0.063	0.054	0.921	0.916	0.916

^a^Intellectual humility leadership and core self-evaluation are combined into one factor; ^b^core self-evaluation and positive attitude in workplace are combined into one factor, thriving at work and work performance are combined into one factor; ^c^Intellectual humility leadership, core self-evaluation, and positive attitude in workplace are combined into one factor, thriving at work and work performance are combined into a second factor.

The descriptive statistics and correlation coefficients among variables are presented in Table 3. According to Table 3, Intellectual humility leadership is significantly positively correlated with positively attitude in workplace among new generation employees (*r* = 0.353, *p* < 0.05), work performance (*r* = 0.204, *p* < 0.05), and thriving at work (*r* = 0.130, *p* < 0.05). Core self-evaluation among new generation employees is significantly positively correlated with positive attitude in workplace (*r* = 0.632, *p* < 0.05), work performance (*r* = 0.677, *p* < 0.05), and thriving at work (*r* = 0.730, *p* < 0.05). Positive attitude in workplace among new generation employees is significantly positively correlated with work performance (*r* = 0.453, *p* < 0.05) and thriving at work (*r* = 0.489, *p* < 0.05). Work performance among new generation employees is significantly positively correlated with thriving at work (*r* = 0.520, *p* < 0.05). There are significant pairwise correlations among the main variables, indicating suitability for regression analysis.

**Table 3:** Correlation analysis.

Variables	Mean	SD	1	2	3	4	5	6	7	8	9	10
Gender	1.47	0.500	1									
Age	2.42	1.070	−0.098*	1								
EA	2.52	0.894	0.044	−0.007	1							
YWE	2.90	1.255	−0.050	−0.024	0.074	1						
IA	11.66	6.269	−0.022	−0.026	0.067	0.014	1					
IH	11.66	6.269	−0.006	0.027	−0.08	0.007	−0.056	1				
CSE	3.591	1.076	−0.076	0.079	0.007	−0.011	−0.034	0.353**	1			
PA	3.547	1.198	−0.021	−0.003	−0.022	0.005	−0.03	0.204**	0.453**	1		
WP	3.569	1.146	−0.008	−0.016	−0.052	−0.05	−0.075	0.130**	0.489**	0.520**	1	
TaW	3.586	1.165	−0.036	0.002	−0.011	0.006	−0.037	−0.073	0.632**	0.677**	0.730**	1

* indicates *p* < 0.1, ** indicates *p* < 0.05. The coefficients in the table are standardized coefficients. The abbreviations used are as follows: Intellectual Humility Leadership (IH), Core Self-Evaluation (CSE), Positive Attitude in Workplace (PA), Thriving at Work (TaW), Work Performance (WP), Gender (Gender), Age (Age), Education Background (EA), Years of Work Experience (YWE), and Industry Affiliation (IA).

### 3.4 Hypothesis testing

#### 3.4.1 Main effects test

As shown in Table 4, after controlling for demographic variables, intellectual humility leadership was found to be significantly positively related to positive work attitudes of new-generation employees (*β* = 0.393, *p* < 0.01), thereby supporting Hypothesis 1 (H1). It was also significantly positively related to thriving at work (*β* = 0.135, *p* < 0.01), supporting Hypothesis 2 (H2), and to job performance (*β* = 0.216, *p* < 0.01), supporting Hypothesis 3 (H3). In addition, positive work attitudes were significantly positively related to both job performance (*β* = 0.437, *p* < 0.01) and thriving at work (*β* = 0.480, *p* < 0.01), thereby supporting Hypotheses 4 (H4) and 6 (H6).

**Table 4:** Main effects test.

Variables		PA		WP			TaW		
		Model 1	Model 2	Model 3	Model 4	Model 5	Model 6	Model 7	Model 8
Control variable	Constant	3.671 **	2.165**	2.165**	2.942**	2.165**	4.106**	3.590**	2.346**
		(12.290)	−6.551	(−7.424)	(−8.837)	(−7.424)	(−14.12)	(−10.515)	(−8.136)
	Gender	−0.169	−0.169	0.025	−0.049	0.025	−0.028	−0.028	0.053
		(−1.596)	(−1.704)	(−0.269)	(−0.493)	(−0.269)	(−0.270)	(−0.272)	(−0.591)
	Age	0.080	0.069	−0.041	−0.012	−0.041	−0.022	−0.026	−0.06
		(1.608)	(−1.498)	(−0.957)	(−0.248)	(−0.957)	(−0.461)	(−0.537)	(−1.436)
	EA	0.019	0.055	−0.033	−0.004	−0.033	−0.057	−0.044	−0.066
		(0.315)	(−0.992)	(−0.642)	(−0.079)	(−0.642)	(−0.986)	(−0.771)	(−1.310)
	SY	−0.013	−0.017	0.01	0.002	0.01	−0.043	−0.045	−0.037
		(−0.307)	(−0.444)	(−0.29)	(−0.059)	(−0.29)	(−1.058)	(−1.104)	(−1.041)
	IA	−0.007	−0.003	−0.003	−0.004	−0.003	−0.013	−0.012	−0.01
		(−0.775)	(−0.395)	(−0.362)	(−0.453)	(−0.362)	(−1.646)	(−1.512)	(−1.450)
Independent variable	IH		0.393**		0.216**			0.135**	
			(−8.545)		(−4.651)			(−2.839)	
Mediator variable	PA					0.437**			0.480**
						(−11.512)			(−12.796)
	$R^{2}$	0.012	0.136	0.002	0.042	0.207	0.011	0.026	0.251
	$\Delta R^{2}$	0.003	0.126	−0.008	0.031	0.198	0.001	0.014	0.242
	*F*	1.281	13.386***	0.187	3.768***	22.285**	1.087	2.261*	28.482***

* indicates *p* < 0.1, ** indicates *p* < 0.05, *** indicates *p* < 0.01. The abbreviations used are as follows: Intellectual Humility Leadership (IH), Core Self-Evaluation (CSE), Positive Attitude in Workplace (PA), Thriving at Work (TaW), Work Performance (WP), Gender (Gender), Age (Age), Education Background (EA), Years of Work Experience (YWE), and Industry Affiliation (IA).

#### 3.4.2 Mediation effect test

In testing the mediation model, this study adopted structural equation modeling (SEM) to examine the indirect effects among the variables. Compared the theoretical model, nested models, and alternative models to identify the best-fit model. Prior to analysis using AMOS 28 software, we conducted parceling of measurement items through a balanced method due to the large number of measurement items; this step resulted in each variable containing 3 items (Wu and Wen, 2011). The theoretical model assumes no direct effect of intellectual humility leadership on work performance and thriving at work among new generation employees; the nested model adds direct effects based on the theoretical model; the alternative model assumes no mediating effect, with intellectual humility leadership, core self-evaluation, and positive attitude in workplace directly influencing work performance and thriving at work among new generation employees.

Firstly, comparing the theoretical model with the nested model: in terms of fit indices, the theoretical model ( $\chi^{2}/\mathrm{df}$ = 2.217; RMSEA = 0.049, SRMR = 0.076, CFI = 0.982, TLI = 0.977) and the nested model ( $\chi^{2}/\mathrm{df}$ = 2.245; RMSEA = 0.049, SRMR = 0.074, CFI = 0.982, TLI = 0.976) both demonstrate good fit. Following Anderson’s recommended method, the change in chi-square (∆ $\chi^{2}/\mathrm{df}$ = 0.028, *p* > 0.05) between the theoretical model and nested model indicates that adding direct paths did not significantly improve the fit of the theoretical model. The alternative model did not fit well, thus confirming that the theoretical model better reflects the data relationships among variables compared to the nested and alternative models.

**Figure 2:**
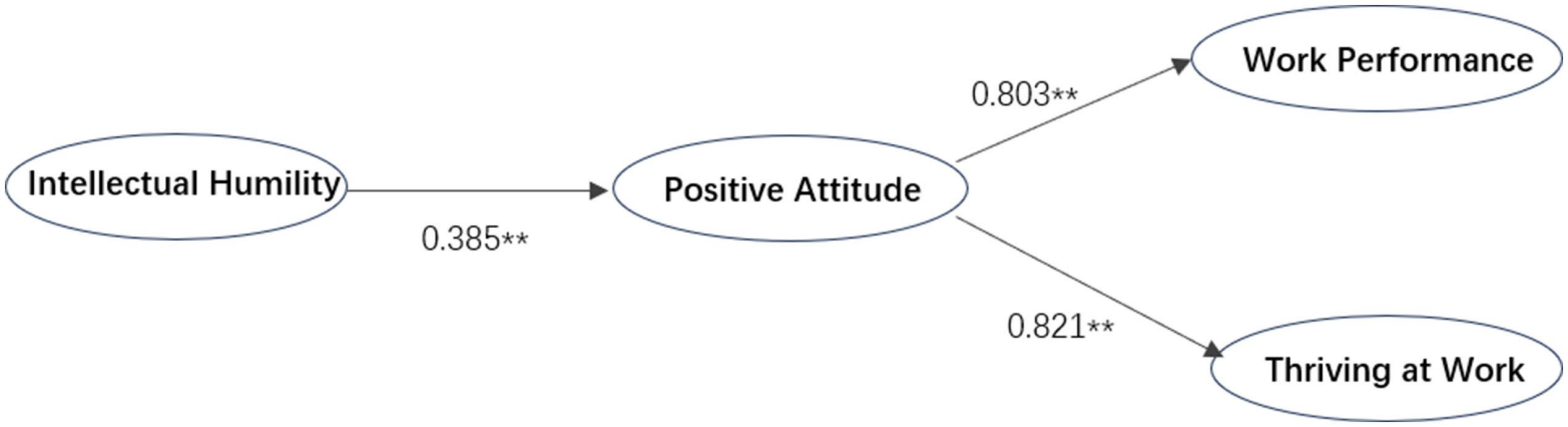
The results of the theoretical model. Note:** indicates *p* < 0.05.

Positive work attitudes had a significant effect on the relationship between intellectual humility leadership and job performance (*β* = 0.163, *p* < 0.01), as well as between intellectual humility leadership and thriving at work (*β* = 0.193, *p* < 0.01), providing preliminary evidence for the mediating role of positive work attitudes. With 2,000 bootstrap samples, the 95% confidence intervals were [0.106, 0.208] and [0.126, 0.231], respectively, both excluding zero, thereby further confirming the significant mediating effect of positive work attitudes. Therefore, Hypotheses 5 (H5) and 7 (H7) were supported. The results of the theoretical model are shown in Figure 2.

#### 3.4.3 Moderated mediation effects test

Using Latent Moderated Structural Equations (LMS), the study tested moderated mediation effects. The results indicate that the interaction term between intellectual humility leadership and core self-evaluation significantly influences positive attitude in workplace among new generation employees (*β* = 0.071, *p* < 0.05), suggesting that core self-evaluation moderates the relationship between intellectual humility leadership and positive attitude in workplace.

As shown in Table 5, the 95% bias-corrected confidence interval of $a_{3}b_{1}$ with 2,000 bootstrap samples was [0.000, 0.062] when job performance was the dependent variable, excluding zero, thereby supporting the moderated mediation hypothesis H8a. When thriving at work was the dependent variable, the interval was [0.000, 0.072], also excluding zero, thereby supporting the moderated mediation hypothesis H8b. These results indicate that the indirect effects of intellectual humility leadership on job performance and thriving at work through positive work attitudes are moderated by core self-evaluation. Specifically, under high levels of employees’ core self-evaluation, the effects of intellectual humility leadership on both thriving at work and job performance through positive work attitudes are stronger than under low levels.

**Table 5:** Moderated mediation effect analysis.

Implicit variable	Moderator variable	Effect	Std. error	Confidence interval	$a_{3}b_{1}$	Std. error	Confidence interval
WP	Low (Mean − 1SD)	0.151	0.025	[0.104,0.203]	0.029	0.016	[0.000,0.062]
	Medium (Mean)	0.183	0.025	[0.137,0.235]			
	High (Mean + 1SD)	0.215	0.025	[0.152,0.288]			
TaW	Low (Mean − 1SD)	0.179	0.028	[0.125,0.235]	0.035	0.018	[0.000,0.072]
	Medium (Mean)	0.217	0.027	[0.167,0.270]			
	High (Mean + 1SD)	0.255	0.038	[0.185,0.331]			

The coefficients in the table are standardized coefficients. The abbreviations used are as follows: Thriving at Work (TaW), Work Performance (WP).

## 4 Discussion

This study extends leadership research by demonstrating how intellectual humility enhances new-generation employees’ thriving and performance. It provides the first empirical evidence in an Asian cultural context showing that intellectual humility leadership influences new-generation employees through the mediating role of positive work attitudes.

### 4.1 The effects of intellectual humility leadership on new-generation employees

The results indicate that intellectual humility leadership has a significant positive impact on both job performance and thriving at work among new-generation employees. This finding aligns with the values of Millennials and Generation Z, who place greater emphasis on autonomy, meaningful work, and personal growth. Leaders who acknowledge their own limitations, remain open to feedback, and embrace diverse perspectives foster an atmosphere of inclusiveness and psychological safety, which stimulates vitality and learning, promotes thriving at work, and ultimately enhances performance. Building on Krumrei-Mancuso and Begin (2022), this study further extends these insights by focusing specifically on new-generation employees. In contrast to transformational leadership, which may risk suppressing dissenting opinions and undermining psychological safety when overly emphasizing the leader’s personal vision (Lin et al., 2020; Eisenbeiß and Boerner, 2013; Harsono et al., 2025), IH leadership actively encourages open dialog and diverse viewpoints (Leary et al., 2017). This inclusive approach not only preserves but also strengthens psychological safety, enabling employees to feel respected and supported even in conflict situations. Such characteristics are particularly important for new-generation employees who pursue self-fulfillment and long-term career development.

From the perspective of the JD-R theory, IH serves as a job resource that, by conveying openness and respect, not only buffers demands but also facilitates motivational processes (Taris and Schaufeli, 2015). From the perspective of SET, IH leadership conveys signals of trust and humility, which employees interpret as fairness and respect. In return, employees reciprocate through stronger commitment, greater effort, and enhanced job performance (Cropanzano and Mitchell, 2005).

### 4.2 The mediating role of positive work attitudes

The results demonstrate that positive work attitudes play a key mediating role between intellectual humility (IH) leadership and employee outcomes (thriving at work and job performance). Once this mediating variable is included, the direct effects of IH leadership become insignificant, indicating that such leadership does not directly influence outcomes but instead operates through shaping employees’ attitudes. This finding underscores that positive work attitudes represent the primary rather than supplementary pathway through which leadership exerts its effects. Theoretically, this result reflects a dual logic of “response” and “exchange”: according to the Job Demands–Resources (JD-R) theory, employees perceive leaders as a job resource and respond with positive attitudes; according to social exchange theory (SET), employees reciprocate leaders’ openness and respect by adopting positive attitudes. For new-generation employees who place high value on autonomy and self-worth, such attitudes are especially critical, as they stimulate vitality, enhance performance, and further amplify the positive effects of IH leadership.

This conclusion is consistent with previous research (Casimir et al., 2014; Abbas et al., 2016), which suggests that leadership behaviors influence employee outcomes through attitudinal mechanisms, thereby confirming the central role of positive work attitudes in linking IH leadership with thriving and performance. In contrast to studies emphasizing that leadership can directly enhance performance (Yukl, 2008), our findings suggest that IH leadership enhances employees’ autonomy and intrinsic motivation, thereby fostering more enduring and stable performance, while simultaneously reducing reliance on external supervision and material incentives, ultimately lowering managerial costs.

### 4.3 The moderated mediation role of core self-evaluation

Research shows that core self-evaluation (CSE) plays a critical moderated mediation role in the mechanism through which intellectual humility (IH) leadership influences employee outcomes. For employees with high levels of CSE, greater confidence, self-efficacy, and a stronger sense of control make them more likely to translate leaders’ openness and inclusiveness into positive work attitudes. As a result, the mediating pathway of IH leadership—positive work attitudes --thriving/performance” is significantly amplified, reflecting a strong moderating effect.

First, according to the Job Demands–Resources (JD-R) theory, CSE, as a stable personal resource, determines the extent to which employees can effectively utilize the job resources provided by leaders (Tummers and Bakker, 2021). Employees with high CSE, due to their abundant personal resources, are better able to mobilize and integrate leaders’ support and autonomy, and thus, through positive attitudes, demonstrate higher levels of thriving at work. In contrast, employees with low CSE, owing to weaker confidence and a limited sense of control, have constrained capacity to mobilize resources, leading to a weaker mediating effect. Nevertheless, because IH leadership fosters psychological safety, respects individual differences, and encourages voice, employees with low CSE can still gradually develop positive attitudes, which in turn promote growth and performance. This finding resonates with Judge and Bono (2001), who noted that CSE significantly shapes how individuals perceive and utilize their organizational environment.

Second, from the perspective of social exchange theory (SET), IH leadership conveys signals of humility and respect, which employees interpret as fairness and benevolence. Based on reciprocal logic, employees respond with positive attitudes. High-CSE individuals, equipped with stronger confidence and regulatory capacity, are more likely to reciprocate through greater effort and dedication, thereby amplifying the impact of positive attitudes on job performance. Low-CSE individuals, although their responses may be weaker, can still exhibit positive attitudes when supported by leadership; thus, the indirect effect is reduced but not eliminated. This result is consistent with Newman et al. (2018), who emphasized that high levels of personal resources enhance the effectiveness of leadership.

## 5 Practical and theoretical implications

This study addresses the pressing question of how leadership fosters both employee thriving and performance, a challenge that has become particularly urgent with the rise of new-generation employees who seek not only material rewards but also well-being and meaningful work. The findings show that intellectual humility leadership plays a central role by influencing employees through psychological resources and social exchange processes. In contrast to earlier studies that often conflated humility with weakness, this research highlights its distinct positive mechanisms and boundary conditions. These insights not only refine the theoretical understanding of leadership effectiveness but also provide organizations with actionable guidance for cultivating openness and inclusiveness in managerial practice.

This study makes several important theoretical contributions. First, drawing on the dual perspectives of JD-R theory and Social Exchange Theory (SET), it reveals the mechanisms through which intellectual humility leadership influences employee outcomes. Specifically, JD-R theory explains how intellectual humility leadership functions as a critical job resource to promote thriving at work, while SET highlights how intellectual humility leadership serves as a positive signal that enhances job performance through reciprocity. Second, the study confirms the mediating role of positive work attitudes in the leadership mechanism. The results suggest that while intellectually humility behaviors can directly improve employee outcomes, their primary effect operates indirectly through positive attitudes; once the mediator is introduced, the direct effect becomes non-significant. This indicates that positive work attitudes constitute a central pathway, thereby enriching the literature on attitudinal mediation in leadership research. Third, the study identifies the moderating role of core self-evaluation (CSE) in the mediating pathway, showing that higher levels of CSE strengthen the indirect effects of intellectual humility leadership on employee outcomes. This finding deepens the understanding of individual differences as boundary conditions in leadership effectiveness. Finally, by distinguishing intellectual humility from general humility and empirically testing its unique mechanisms (Davis et al., 2016), this study further advances the theoretical conceptualization of intellectual humility in leadership research.

This study also provides valuable practical implications for organizational management. The findings indicate that intellectual humility leadership significantly enhances employees’ attitudes and outcomes, suggesting that organizations should emphasize the cultivation of cognitive openness and inclusiveness in leadership development, rather than focusing solely on modest behavior. Moreover, managers should take into account differences in employees’ CSE when applying leadership strategies, offering differentiated support and developmental opportunities to employees with different characteristics in order to improve management effectiveness and stimulate engagement. In addition, given the mediating role of positive work attitudes, organizations should strive to foster supportive environments that align with employees’ values and provide opportunities for growth, thereby enhancing positivity at work. Finally, it is important to recognize that intellectual humility leadership is only one factor that promotes performance and thriving. In practice, organizations should combine it with job enrichment, psychological empowerment, and career development initiatives to build a systematic human resource management model that achieves win–win outcomes for both employees and organizations.

### 5.1 Limitations

This study still has several limitations that need to be addressed in future research. First, the sample was mainly drawn from certain regions in China, which may lead to insufficient representativeness. Future research could expand the sample scope and further examine cross-cultural and generational differences, as employees from different cultural backgrounds and demographic characteristics may perceive and respond to intellectual humility leadership differently. Second, this study only considered positive work attitudes as the key mediating variable. Future studies could extend the scope to include job satisfaction, organizational commitment, or employee engagement. According to Social Exchange Theory (SET), these factors may provide a more comprehensive explanation of how intellectual humility leadership influences employee attitudes and behaviors. Third, future research should further explore the interaction between intellectual humility leadership and other leadership styles, such as transformational, paternalistic, and contingent leadership, to reveal how different combinations of leadership styles affect employees’ thriving at work and job performance.

## 6 Conclusion

Intellectual humility leadership has recently attracted increasing scholarly attention as an innovative and meaningful topic in leadership research (Krumrei-Mancuso and Begin, 2022). Rooted in leaders’ awareness of their own limitations and openness to diverse perspectives, this leadership style is particularly important in the context of managing Millennials and Generation Z, who value autonomy, voice, and developmental opportunities. The findings of this study demonstrate that intellectual humility leadership exerts a significant positive impact on both thriving at work and job performance among new-generation employees. Moreover, positive work attitudes function as a key mediating mechanism, while core self-evaluation (CSE) strengthens this indirect pathway, thereby supporting a moderated mediation model. Overall, the study concludes that intellectual humility leadership operates not only as a critical resource that enhances employees’ psychological safety and vitality but also as a signal of fairness and support that stimulates reciprocity. In this way, it achieves a win–win outcome for both employees and organizations. Theoretically, it enriches leadership research by extending JD-R theory and refining SET, while practically, it offers useful guidance for cultivating intellectually humility leadership and tailoring management strategies to diverse employee characteristics.

## Data Availability

The raw data supporting the conclusions of this article will be made available by the authors, without undue reservation.
